# Knockdown of long non-coding RNA linc00511 suppresses proliferation and promotes apoptosis of bladder cancer cells via suppressing Wnt/β-catenin signaling pathway

**DOI:** 10.1042/BSR20171701

**Published:** 2018-08-29

**Authors:** Jun Li, Yan Li, Fandong Meng, Liye Fu, Chuize Kong

**Affiliations:** 1 Department of Urology, The First Hospital of China Medical University, Shenyang 110001, China; 2 Institute of Urology, China Medical University, Shenyang 110001, China; 3 Department of Biotherapy, Cancer Research Institute, the First Affiliated Hospital of China Medical University, Shenyang 110001, China

**Keywords:** apoptosis, bladder cancer, LINC00511, proliferation, Wnt/beta-catenin

## Abstract

More and more studies have shown that long non-coding RNAs (lncRNAs) play critical roles in various biological processes of bladder cancer, including proliferation, apoptosis, migration and cell cycle arrest. LncRNA long intergenic noncoding RNA 00511 (linc00511) is one of the lncRNAs highly expressed in bladder cancer tissues and cells. However, little is known about the roles and mechanisms of linc00511 in bladder cancer. Here, we demonstrated that linc00511 was highly expressed in bladder cancer tissues and cells. Linc00511 knockdown could cause the cell proliferation suppression and cell cycle arrest, which were mediated by p18, p21, CDK4, cyclin D1 and phosphorylation Rb. Suppressed linc00511 could induce the apoptosis in T24 and BIU87 cells via activating the caspase pathway. Down-regulation of linc00511 could also suppress the migration and invasion of T24 and BIU87 cells. In addition, we found that the expression of linc00511 was negatively correlated with that of miR-15a-3p in the clinical bladder cancer samples. Further mechanistic studies showed that linc00511 knockdown induced proliferation inhibition, and apoptosis increase might be regulated through suppressing the activities of Wnt/β-catenin signaling pathway. Thus, we revealed that knockdown of linc00511 suppressed the proliferation and promoted apoptosis of bladder cancer cells through suppressing the activities of Wnt/β-catenin signaling pathway. Moreover, we suggested that linc00511 could be a potential therapeutic target and novel biomarker in bladder cancer.

## Introduction

Bladder cancer is one of the most common malignancies of urinary system with the ninth incidence rate among the malignant tumors diagnosed worldwide and second leading mortality rate in male genitourinary tumors [1]. Although ~80% of cases were early diagnosed as non-muscle-invasive bladder cancer, the proportion of recurrent patients in 5 years still accounted for ~60% [2]. Many patients future developed into muscular invasion urothelial carcinomas with a very lower 5-year survival rate. Therefore, it is of great importance to look for a therapeutic target to improve the survival rates of patients with bladder cancer.

Long non-coding RNAs (lncRNAs) have been reported playing critical regulatory roles in pathological and physiological processes of various human diseases including carcinogenesis through regulating the epigenetic inheritance of gene expression, transcription and post-transcriptional levels [3–6]. What’s more, lncRNAs seemed to be involved in various stages of cancer development, including tumor initiation, progression and metastasis, and the disorder of lncRNAs was considered to be the main characteristic of some human cancers [5]. Many known lncRNAs have been observed in the development of cancer and tumor inhibition, such as HOTAIR and MEG3, showing the potential applications of lncRNAs in clinical diagnosis, prognosis, and similar to microRNAs and mRNA treatment [7,8].

So far, numerous studies have showed that lncRNAs are involved in bladder cancer development and progression, such as PVT1 [9], ZEB2NAT [10], MDC1-AS [11] and growth arrest-specific 5 (GAS5) [12]. Zhuang et al*.* [9] reported that PVT1 played the role as an oncogene in bladder cancer by promoting cell proliferation and suppressing apoptosis. The overexpression of ZEB2 regulated by lncRNA ZEB2NAT at the post-transcriptional level could induce EMT in bladder cancer, which predicted the poor clinical outcome [10,13]. LncRNA MDC1-A down-regulated in bladder cancer tissues could inhibit the malignant phenotype of bladder cancer cells by up-regulated MDC1 expression [11]. GAS5 down-regulated in bladder cancer tissues could suppress the proliferation of bladder cancer cells and enhance cancer cell apoptosis [12]. However, the detailed molecular mechanisms of lncRNAs in bladder cancer still require further clarified.

Long intergenic noncoding RNA 00511 (linc00511), as an oncogenic lncRNA, could drive the tumorigenesis in NSCLC by regulating the cell proliferation, apoptosis, invasion and metastasis [14]. The similar results could also find in pancreatic ductal adenocarcinoma that the increase in linc00511 indicated the poor pathological features and prognosis [15]. However, little is known about the effects of linc00511 on the underlying molecular mechanisms and the biological roles in bladder cancer. Here, we explored the functional and molecular characterization of linc00511 in bladder cancer. Our data showed that linc00511 expression was higher in bladder cancer patients than in healthy individuals, the similar results were also found in bladder cancer cells. Functionally, knockout of linc00511 could significantly inhibit the proliferation and promote apoptosis of bladder cancer. The associations of linc00511 expression with the miR-15a-3p and Wnt/β-catenin signaling pathway were also assessed. In addition, we provided the evidences that knockdown of linc00511 suppressed the proliferation and promoted apoptosis of bladder cancer via suppressing Wnt/β-catenin signaling pathway.

## Materials and methods

### Tissue samples

In the present study, the bladder cancer and the non-cancerous tissues (5 cm from the tumor) from The First Hospital of China Medical University were collected. After resection, all the samples immediately immersed in TRIzol (Takara, China), shattered by an efficient tissue sample processor, and stored at −80°C in order to avoid the degradation of RNA. The tumor grade and stage were available for these samples. Prior to the use of these clinical materials, the written consents of all patients and the approval of The First Hospital of China Medical University Ethic Review Committees had been obtained.

### Cell culture and transfection

Human urothelial cell line (SV-HUC-1) and human bladder cancer cell lines (BIU87, T24 and 5637) were obtained from the American Type Culture Collection (ATCC). SV-HUC-1 cells were cultured in F-12K Medium (Gibco, U.S.A.), while others were cultured in RPMI-1640 medium (Gibco, U.S.A.) supplemented with 10% fetal bovine serum (Gibco, U.S.A.) and penicillin/streptomycin (100 U/ml and 100 μg/ml respectively, HyClone) at 37°C in an atmosphere of 5% CO_2_. Si-linc00511 was purchased from GenePharma (Shanghai, China). Cells were cultured with complete medium without antibiotics at least 24 h prior to transfection, then washed with PBS and transiently transfected with 50 nmol/l si-linc00511 and si-NC, using Lipofectamine 2000 (Invitrogen, CA) according to the manufacturer’s instructions.

### RNA isolation and quantitative real-time PCR

Total RNA from clinical samples and cultured cell lines were extracted by TRIzol (Takara, China), then reverse transcribed using PrimeScript™ RT Master Mix (Takara, China) according to the manuals. The levels of linc00511, miR-15a-3p, cyclin D1, c-myc, β-catenin and GAPDH were assessed using SYBR^®^ Premix Ex Taq™ (Takara, China) and conducted on an Applied Biosystems Prism 7500 Fast Sequence Detection System (Applied Biosystems, U.S.A.). GAPDH was used as the quantitative control. Quantitative PCR parameters for cycling were as follows: start at 95°C for 5 min, 40 cycles of PCR at 95°C for 3 s, 60°C for 30 s and 72°C for 30 s. The primers for real-time PCR were as follows: linc00511 sense: 5′-CGCAAGGACCCTCTGTTAGG-3′, antisense: 5′-GAAGGCGGATCGTCTCTCAG-3′; cyclin D1 sense: 5′-GTCTTCCCGCTGGCCATGAACTAC-3′, antisense: 5′-GGAAGCGTGTGAGGCGGTATAGG-3′; c-myc sense: 5′-TCAAGAGGTGCCACGTCTCC-3′, antisense: 5′-TCTTGGCAGCAGGATAGTCCTT-3′; β-catenin sense: 5′-CAGAAGCTATTGAAGCTGAGG-3′, antisense: 5′-TTCCATCATGGGGTCCATAC-3′; GAPDH: sense: 5′-CTCTGCTCCTCCTGTTCGAC-3′, antisense: 5′-ACCAAATCCGTTGACTCCGA-3′. The experiments were repeated at least three times, and each sample was also tested in triplicate. The formula and its derivations were obtained from the ABI Prism 7500 sequence detection system user guide.

### MTT cell proliferation assay

The effects of linc00511 on cell growth in T24 and BIU87 cells were measured using a MTT Cell Proliferation Assay Kit (Sigma, U.S.A.). A total of 1000 cells transfected with si-linc00511 or si-NC per well were seeded in the 96-well plates. Add MTT to the three repeat wells and measure the absorbance at 490 nm every 24 h for 4 days. The absorbance values were the ordinate of cell growth curves, while times were as the *x*-coordinate.

### Colony formation assay

T24 and BIU87 cells transfected with si-linc00511 or si-NC were plated at a density of 400 cells per well in a 6-well plate (corning, U.S.A.), then cultured in medium supplemented with 10% FBS, which was refreshed every three days. Cell colonies were allowed to grow for 7 days before staining with 0.1% Crystal Violet (Sigma, U.S.A.) solution. The experiments were repeated at least three times.

### Knockdown of linc00511 expression

Small interfering RNAs (siRNAs) targeting linc00511 (5′-CCCAUGUCUGCUGUGCCUUUGUACU-3′) or a control (a scrambled matched %GC oligonucleotide, Invitrogen) were used (GenePharma, Shanghai, China) in the transient transfection experiment. T24 and BIU87 cells were seeded at a density of 10^5^ cells per well in 6-well plates. The cells were transfected with siRNAs by Lipofectamine 2000 (Invitrogen, CA) according to the manufacturer’s instructions after 24 h. Knockdown efficiency was assessed with quantitative real-time PCR (qPCR) assay.

### EdU assay

Thymidine analog 5-ethynyl-2′-deoxyuridine (EdU) staining was used to assess the cell proliferation. Cells were seeded in 24-well plates and transfected with si-linc00511and si-NC. The EdU staining was used to detect cell proliferation according to manufacturer’s instructions after transfection for 48 h. In short, the cells were incubated with 50 μM EdU for 2 h before fixation, permeabilization and EdU staining, all of these executed according to the manufacturer’s protocol. The DAPI (Sigma-Aldrich) stained cell nuclei was at a concentration of 1 mg/ml, 10 min. The proportion of EdU stained cells was determined by fluorescence microscopy.

### Cell migration and invasion assay

The migration capacity of cells was examined by wound healing assay. A total of 5 × 10^5^ cells were seeded in 6-well culture plates after transfection for 48 h and grown to ~100%. The cell sheet was vertically wounded by a 10-μl tip and then washed with PBS for three times to remove the cell debris. ImageJ software was used to measure the wound width at the designated time periods.

The tumor cell invasion capacity was measured by transwell assay. T24 and BIU87 cells were transfected with si-linc00511and si-NC for 48 h, then suspended in RPMI-1640 medium at a final density of 1 × 10^6^/ml. Suspensions (100 μl) were seeded into the upper chamber that had a porous membrane coated with Matrigel (BD Biosciences, U.S.A.), and 500 μl of 10% FBS containing medium was added to the bottom chamber. Then the cells were cultured for 48 h, and the migrated cells were fixed in methanol, stained in Crystal Violet. The numbers of invasive cells were counted from six random fields under a microscope (Nikon, Japan).

### *In vivo* xenograft experiments

Male BALB/c nude mice (6-week-old; *n*=6) were purchased from Beijing HFK Bioscience Co. Ltd. (Beijing, China) and maintained under pathogen-free conditions with approval by the Committee of The First Hospital of China Medical University. For tumor propagation analysis, 1 × 10^7^ T24 tumor cells were subcutaneously injected into BALB/c nude mice. Tumor volume was calculated using the formula: volume = π*ab*^2^/6 (*a*, tumor length; *b*, tumor width) at indicative time points. Tumor weight was measured at week 5 post-injection. Animal experiments were performed in accordance with relevant guidelines and regulations of the Animal Care and Use Committees at the The First Hospital of China Medical University, and a signed document issued by the Animal Care and Use Committees that granted approval was obtained.

### Protein extraction and Western blotting

The T24 and BIU87 cells transfected with si-linc00511 and si-NC were washed third by cold PBS and then lysed using the RIPA buffer (Thermo Scientific, U.S.A.) containing phenylmethanesulfonyl fluoride. Proteins (40 μg per sample) separated by 10% SDS-PAGE and then transferred electrophoretically onto a PVDF membrane. Blocked the membranes in 5% BSA diluted in TBST, then incubed with appropriate antibodies (CDK4, p18, cyclin D1, p21, p-Rb, Rb, poly (ADP-ribose) polymerase (PARP), caspase-3, caspase-9, β-catenin, cyclin D1 and c-myc) and GAPDH (Cell Signaling Technology, 1:1000) overnight at 4°C. Then, the membranes were washed for three times with TBST, and immediately following incubated with HRP-conjugated goat anti-rabbit secondary antibodies (Cell Signaling Technology, 1:1000) for 1 h at room temperature. GAPDH was used as the internal control. The bands were visualized using an enhanced chemiluminescence kit (ECL, U.S.A.) and used the X-ray film to autoradiography.

### Flow cytometry assay

The flow cytometry analysis was used to determine whether the silence oflinc-00511 could inhibit the growth phase of T24 and BIU87 cells. T24 and BIU87 cells transfected with si-NC or si-linc00511 were harvested at 48 h and resuspended at 1 × 10^6^ cells/ml, then dyed annexin V-FITC and propidium iodide (PI) according to the manufacturer’s instructions. The reaction was analyzed with FACSCalibur by using the Cell Quest software (Becton Dickinson, U.S.A.). Cells were fixed with 70% ice-cold ethanol and labeled with PI in the growth phase analysis. The cell cycle was analyzed using Cell Quest software.

### Statistical analysis

The statistical analysis was performed using GraphPad Prism 5.0 (GraphPad Software, U.S.A.). The differences of the results were carried out by fold change and analyzed by the Student’s *t*-test.

## Results

### Linc00511 expression increased in human bladder cancer tissues and cell lines

**Figure 1:**
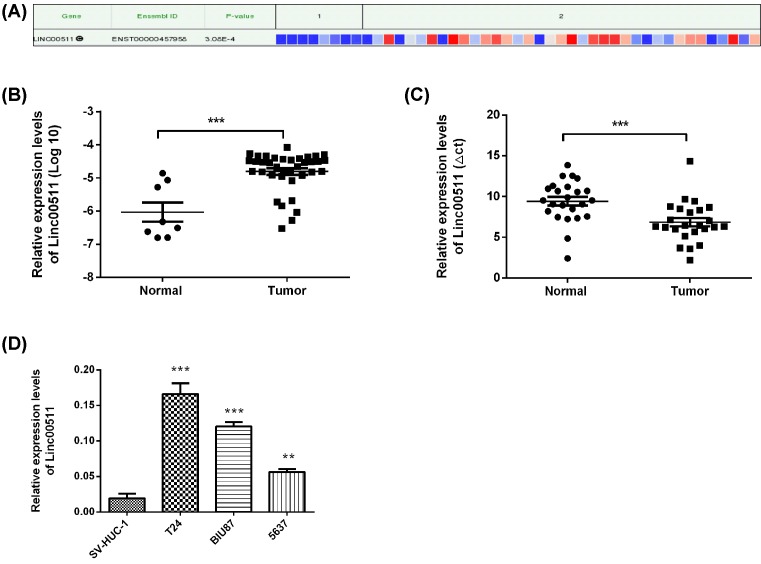
Linc00511 expression increased in human bladder cancer tissues and cell lines (**A**) The bioinformatics tools lncRNAtor were used to analyze the RNA-Seq data from TCGA for lncRNAs in bladder cancer tissues (http://lncrnator.ewha.ac.kr/index.htm). Normal tissues (*n*=8) and tumor tissues (*n*=37). (**B**) The analysis of linc00511 expression in bladder cancer tissues (*n*=37) was used TCGA database, compared with adjacent non-tumor tissues (*n*=8). (**C**) qPCR was also used to examine the relative linc00511 expression in bladder cancer tissues (*n*=24) compared with the adjacent non-tumor tissues (*n*=24). (**D**) The expression levels of linc00511 in various bladder cancer cells and in SV-HUC-1 cells were analyzed by qPCR. ***P<0.001, **P<0.001 compared with Normal or SV-HUC cell line.

The differences of linc00511 expression in bladder cancer and adjacent tissues (*n*=45) were analyzed by the bioinformatics tool lncRNAtor (http://lncrnator.ewha.ac.kr/index.htm). The results showed that the expression levels of linc00511 were significantly increased in bladder cancer tissues (Figure 1A). Moreover, the expression of linc00511 in 45 bladder cancer tissues and non-tumorous adjacent tissues from TCGA was also analyzed (Figure 1B), consistent with the results of Figure 1A (*P*<0.01). Moreover, the linc00511 expression levels in 24 pairs of bladder cancer tissues and non-tumorous adjacent tissues were also detected by qPCR. The results showed that the expression levels of linc00511 in tumors were much higher than the corresponding normal tissues (Figure 1C). Then, the expression levels of linc00511 in SV-HUC-1, BIU87, T24 and 5637 were examined by the qPCR analysis. BIU87, T24 and 5637 cancer cells showed the higher expression of linc00511 than that of SV-HUC-1 cells (Figure 1D). Therefore, the effects of linc00511 on cell physiological behaviors were studied using the BIU87 and T24 cancer cells as the model.

### Linc00511 knockdown inhibited the proliferation of BIU87 and T24 cells

**Figure 2:**
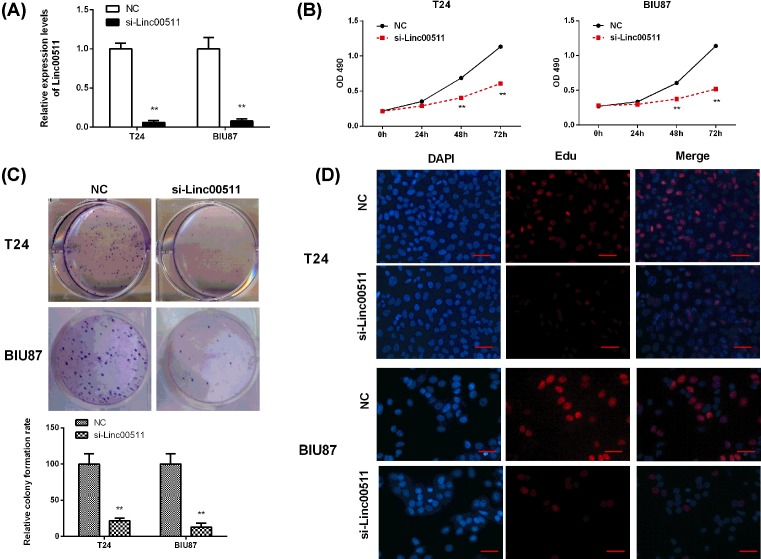
Linc00511 knockdown inhibited the proliferation of BIU87 and T24 cells (**A**) The knockdown efficiency of linc00511 in T24 and BIU87 cells by transfected with si-linc00511 (*n*=3). (**B**) MTT analysis showed that the knockout of linc00511 destroyed cell proliferation in T24 and BIU87 cells (*n*=6). (**C**) Histological analysis of colony formation rates in si-NC and si-linc00511 groups (*n*=6). (**D**) The effects of linc00511 inhibition on DNA synthesis were examined by EdU assay during cell growth (×200). The results showed that the proportion of S-phase cells was decreased in si-linc00511 treated groups (*n*=6). **P<0.01 compared with NC.

To investigate the effect of linc00511 on the proliferation of bladder cancer cells, the expression of linc00511 was knocked down in BIU87 and T24 cancer cells by transfection with si-linc00511. The interfering efficiencies of linc00511 in BIU87 and T24 cancer cells detected by qPCR were both ~90%, proving the significant interference effect and beneficial to the follow-up experiment (*P*<0.05, Figure 2A).

These results displayed that linc00511 knockdown could sharply reduce the proliferation in BIU87 and T24 cells after cultured for 48 h, but there was no significant effect at 24 h, as revealed by MTT assay (Figure 2B). The proliferation rates of BIU87 and T24 cells were also significantly reduced after transfection with si-linc00511 compared with the si-NC groups (*P*<0.05). Consistent with the above results, the clone formation abilities of BIU87 and T24 cells also significantly suppressed after knockdown of linc00511 when compared with those negative controls (*P*<0.05, Figure 2C). The EdU assay was also used to detect the ability of linc00511 to inhibit the DNA synthesis during cell growth. The results showed that the proportion of S-phase cells decreased in si-linc00511 treated cells compared with si-NC groups, indicating that linc00511 depletion resulted in DNA synthetic activity (Figure 2D). All the above results proved that linc00511 depletion had a significant inhibitory effect on the growth of bladder cancer cells.

### Knockdown of linc00511 resulted in the intrinsic apoptosis and cell cycle arrest in bladder cancer cells

**Figure 3:**
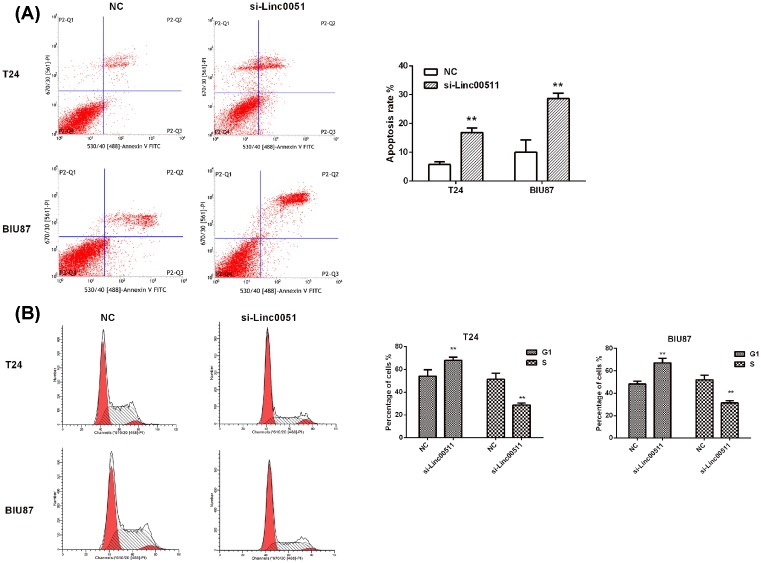

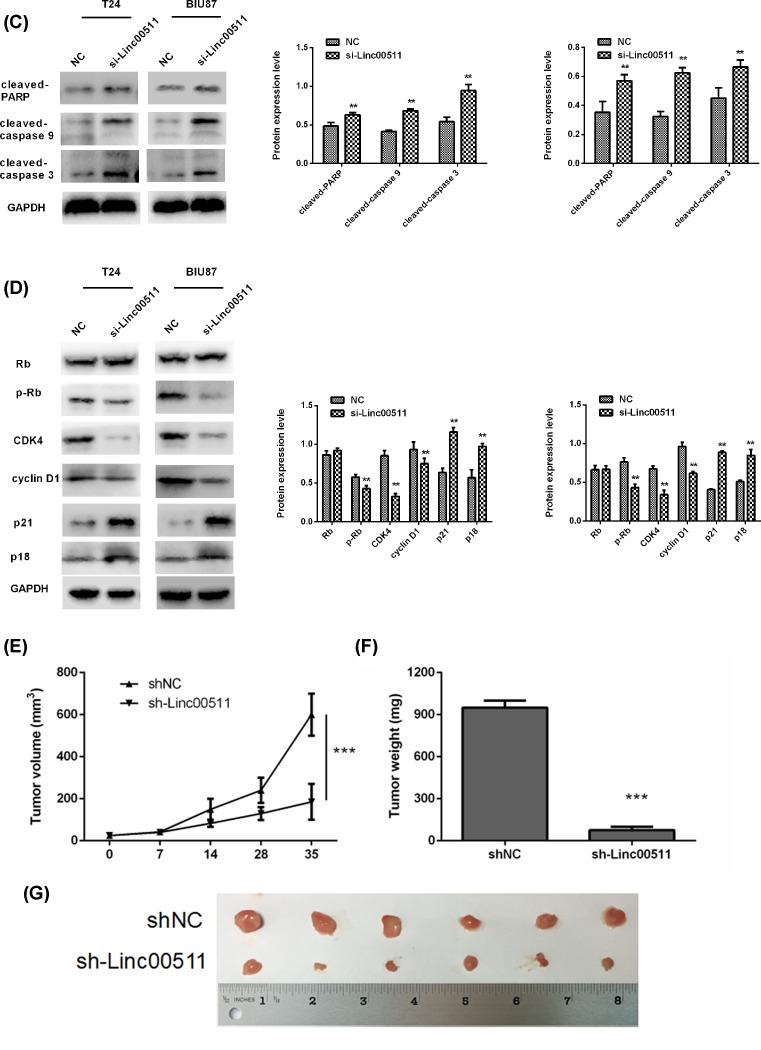
Knockdown of linc00511 resulted in the intrinsic apoptosis and cell cycle arrest in bladder cancer cells (**A**) The T24 and BIU87 cells stained with annexin V-FITC/PI and analyzed by flow cytometry after transfection for 48 h. (**B**) Cell cycle of T24 and BIU87 cells were detected by flow cytometry after transfection with si-linc00511 for 48 h. The percentage of G1 and S-phase cells was showed. (**C**) The changes of apoptosis-related proteins in T24 and BIU87 cells were detected by Western blot. (**D**) The alterations of cell cycle-related proteins in bladder cancer cells with linc00511 knockdown. **P<0.01 compared with NC. (**E**) Tumor growth curves were established by measuring tumor volume every 7 for 35 days after injection. (**F–G**) Tumor weights isolated from nude mice in each treatment group were determined on day 35 after injection. ***P<0.001.

The involvements of linc00511 in the cell apoptosis and cell cycle were also detected by knockdown of linc00511 expression. Si-linc00511 treatment caused a significant increase in the rate (5.725 ± 0.86% and 10.001 ± 2.93% in si-NC groups to 16.865 ± 1.19% and 28.660 ± 1.57% in si-linc00511 groups) of apoptosis in T24 and BIU87 cells respectively (Figure 3A, *P*<0.01). Moreover, the activities of cleaved caspase-3 and cleaved caspase-9, as the prominent markers of caspase pathway, were significantly increased when transfected with si-linc00511 (Figure 3C, *P*<0.05). The expression of PARP also showed the similar results (Figure 3C, *P*<0.05). These findings indicated that the apoptosis-induced bysi-linc00511 treatment was at least partly through the activation of the intrinsic apoptotic pathway. The results of cell cycle analysis showed that the percentage of cells in the G1 phase was increased from 48.2 ± 2.98% and 54.025 ± 4.37% in si-NC groups to 67.13 ± 3.17% and 68.065 ± 2.37% in si-linc00511 groups in T24 and BIU87 cells, respectively, the percentage of cells in the S phase decreased at the same time (*P*<0.05, Figure 3B). The expressions of cell cycle regulating proteins induced by linc00511 were also detected by Western blot after transfection with si-linc00511 for 48 h, the results showed knockdown of linc00511 could lead to a significantly increased expression of p18 and p21, a sharply decreased expression of cyclin D1, CDK4, Rb in T24 and BIU87 cells (*P*<0.05, Figure 3D). In addition, the biological effects of linc00511 on bladder cancer development were evaluated in a xenograft mouse model. T24 cells transfected with shNC or shlinc00511 were implanted subcutaneously into nude mice. Then tumor growth was evaluated every 7 days. Linc00511 knockdown significantly delayed tumor growth *in vivo* (Figure 3E,F). At 5 weeks post-implantation, the nude mice were killed, and tumors were harvested and weighed. LINC01296 knockdown significantly decreased the tumor size and weight (Figure 3G). These results indicated that linc00511 knockdown resulted in the cell cycle arrest in G1 phase, which might be a reason for the suppressed proliferation.

### The migration and invasion in bladder cancer cells were inhibited by linc00511 knockdown

**Figure 4:**
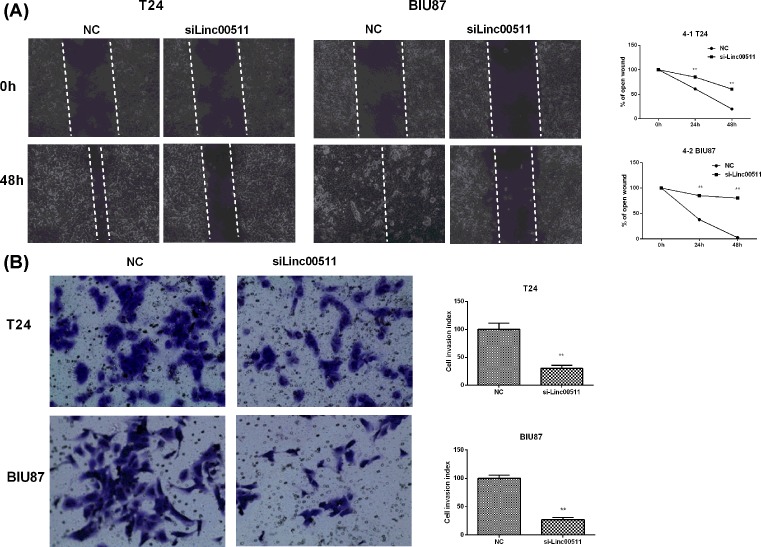
Knockdown of linc00511 inhibited the migration and invasion in bladder cancer cells (**A**) Wound healing assays were used to demonstrate that the migratory potential of T24 and BIU87 cells was significantly reduced by si-linc00511 transfection when compared with si-NC groups. (**B**) Matrigel invasion assay results showed that the invasion potential of linc00511-silenced T24 and BIU87 cells was sharply reduced when compared with si-NC groups. **P<0.01 compared with NC.

The motility and invasion abilities were crucial to the progression and metastasis of cancer. Therefore, the role of linc00511 knockdown in bladder cancer cells migration was explored by the wound healing assay. The BIU87 and T24 cells treated with si-linc00511 showed a significant slower motility (the relative proportion of wound closure) compared with the si-NC groups (Figure 4A). Furthermore, the migration and invasion abilities of BIU87 and T24 cells were also investigated by matrigel invasion assay when linc00511 expression was suppressed (Figure 4B). The results showed that knockdown of linc00511 expression dramatically reduced the invasion of BIU87 and T24 cells. All these results indicated that linc00511 promoted the migration and invasion of bladder cancer cells.

### Linc00511 was associated with miR-15a-3p

**Figure 5:**
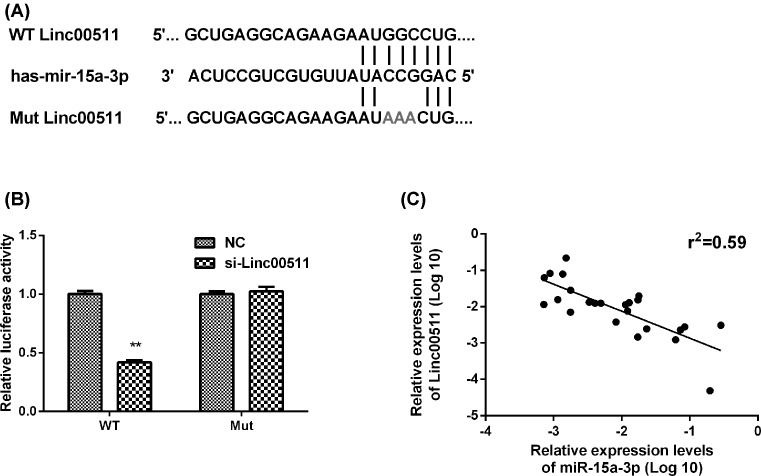
Linc00511 was associated with miR-15a-3p (**A**) Prediction for miR-15a-3p-binding elements on linc00511. The red nucleotides (target sites) were deleted in the mutant constructs. (**B**) The luciferase activity in BIU87 cells were detected by co-transfected with miR-15a-3p and luciferase reporters containing si-NC, si-linc00511 and si-linc00511-mut. Data are presented as the relative ratio of luciferase activity to Renilla luciferase activity. (**C**) RT-PCR detection of miR-15a-3p expression, showing the negative correlation between linc00511 and miR-15a-3p expression levels in bladder cancer tissues (Spearman’s correlation, *r*^2^ = 0.59). **P<0.01 compared with NC.

Previous reports have suggested that lncRNAs could combine with specific miRNAs to interfere with their functions [16]. The bioinformatic analysis revealed the hypothetical complementary sequences for miR-15a-3p in linc00511 (Figure 5A). The luciferase reporter assays were used to further validate the regulatory relationship between miR-15a-3p and linc00511. The full-length linc00511 transcript and mutant linc00511 transcript were cloned into the 3′**-**UTR of *Renilla* luciferase (Rluc) gene of psiCHECK2, which had a downstream constitutively expressed firefly luciferase gene. Results showed that the co-transfection of miR-15a-3p and linc00511 reduced the luciferase activity but not that of miR-15a-3p and mutant linc00511 (Figure 5B). Moreover, the qPCR method was used in bladder cancer tissues to demonstrate that the levels of miR-15a-3p and linc00511 were negatively correlated by linear regression analysis (Figure 5C). The above observations showed that miR-15a-3p expression was negatively correlated with linc00511.

### Knockdown of linc00511 suppressed Wnt/β-catenin signaling activity

**Figure 6:**
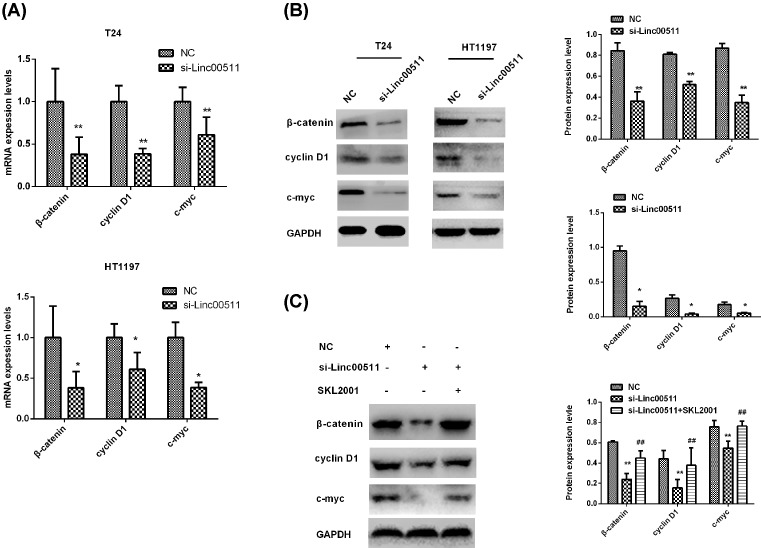
Knockdown of linc00511 suppressed Wnt/β-catenin signaling activity (**A**) The mRNA levels of β-catenin and the downstream genes c-Myc, Cyclin D1 were determined by qPCR after treatment with si-linc00511 in T24 and BIU87 cells. (**B**) The protein levels of c-Myc, Cyclin D1, β-catenin and GAPDH were determined by Western blot assay after treatment with si-linc0051 36 h in T24 and BIU87 cells. (**C**) The changes of c-Myc, Cyclin D1 and β-catenin protein levels in BIU87 cells after treatment with si-linc00511 and si-linc00511 + SKL2001 were also detected by Western blot assay. **P<0.01 compared with NC; ^##^*P*<0.01 compared with si-linc00511. *P<0.05

The Wnt/β-catenin signaling pathway plays an important role innumerous pathological processes including carcinogenesis [17]. To study the effect of linc00511 on Wnt/β-catenin signaling pathway, we detected the activity of Wnt/β-catenin signaling pathway by qPCR and Western blot in BIU87 and T24 cells after transfection with si-linc00511. The results showed that the mRNA and proteins levels of c-Myc, Cyclin D1 and β-catenin were greatly inhibited in si-linc00511 groups compared with si-NC groups (Figure 6A,B). Therefore, we assumed that the Wnt/β-catenin signaling pathway activation might be regulated by linc00511 and the SKL2001 was used as the activator of Wnt/β-catenin signaling pathway. Then, BIU87 cells were treated with si-NC, si-linc00511 and si-linc00511+ SKL2001, respectively. We found that the activity of Wnt/β-catenin signaling pathway reduced by the knockdown of linc00511 expression could reverse by SKL2001 (Figure 6C).

## Discussion

There has been no significant improvement in the treatment of local or metastatic bladder cancer over the past two decades [18], due to the unrestricted proliferation, apoptosis and metastasis of cancer cells. Therefore, it was urgently desired to identify a new molecular target for bladder cancer. More and more studies have showed that several lncRNAs are directly involved in the tumor occurrence and metastasis of different types of cancer, which used as new targets for cancer treatment. In the present study, we have identified the linc00511 as potential molecular targets for bladder cancer treatment.

Linc00511, a newly discovered oncogenic lncRNA, has found highly expressed in breast cancers [19,20], and the silence of linc00511 showed tumor-suppressive functions through inhibiting the proliferation of breast cancer cells [19]. Here, we hypothesized that linc00511 might be involved in the progress of the bladder cancer. In our study, linc00511 was identified as an oncogenic lncRNA through the analysis of 24 pairs of bladder cancer tissues and non-tumorous adjacent tissues, the results showed that linc00511 was up-regulated in bladder cancer tissues. The similar results were also found in bladder cancer cell lines. These results indicated that linc00511 might be a diagnostic biomarker in bladder cancer. However, the correlation between linc00511 expression levels and the tumor histology grade and N grade remains unclear, which needs to be further explored.

The role of linc00511 was further studied by detecting changes in biological behaviors in bladder cancer cells via linc00511 expression silence. The silence of linc00511 resulted in the proliferation suppressed in T24 and BIU87 cells, accompanied with the cell cycle arrest, apoptosis and metastasis suppress. All these results further demonstrated that linc00511 acted as an oncogene in bladder cancer cells.

The progression of cancer is usually associated with the disruption of cell cycle control, which results in the unlimited proliferation of cancer cells [21,22]. The transition from G1 phase to S phase in the cell cycle plays a key role in the proliferation of cells. Here, the knockdown of linc00511 induced cell cycle arrest at G1 phase and reduced the percentage of cancer cells in the S phase through the flow cytometry analysis and EdU assay. As the cyclin D1 could activate the CDK4, promote the phosphorylation of Rb and result in cell transition through G1 phase [23,24], we detected these cell cycle regulating proteins by Western blot. Our findings were consistent with the results obtained from flow cytometry assay in which a decreased level of CDK4–cyclin D complex with the down-regulated phosphorylation of Rb could induce the cell cycle arrest at G1 phase [25]. These results indicated that the cell cycle arrest partly attributed to the anticancer effect of linc00511 knockdown on tumor growth.

Taken into account that defect in apoptosis could cause cancer, the caspase family is essential to the process of apoptosis [26], we detected the activities of the initiator caspase-9 and the effector caspase-3 by Western blot. Our findings showed that the activation of caspase-3 and caspase-9 was induced by the knockdown of linc00511, indicating that the linc00511 might be a therapeutic target for bladder cancer treatment once again. Cancer metastasis is the leading cause of death in cancer patients [27]. Hence, we demonstrated that knockdown of linc00511 expression sharply decreased the migration and invasion ability of T24 and BIU87 cells. However, we had not studied the relevant molecular mechanisms, which will be our next work.

MiRNAs are involved in the regulation of different cellular processes associated with cancer, such as cell proliferation, apoptosis deregulation, deterioration and metastasis of cancer [28,29]. Studies have reported that miR-15a-3p could trigger the activation of apoptosis in tumors by affecting Bcl-xl, thus mediating the activation of caspase-3/7 or promoting the sensitivity of selected cancers to radiotherapy and chemotherapy by the ectopic expression [30–32]. Tao wang et al. [33] reported that miR-15a-3p was an important regulatory miRNA in the tumors EMT process via targeting the Twist1 gene to inhibit the GC cell migration and invasion. Given the importance of miR-15a-3p in tumor suppression and progress, we conducted the luciferase assay by inserting the full-length linc00511 transcript into the luciferase reporter, and found that the co-transfection of miR-15a-3p and linc00511 showed lower luciferase activity and the effect evidently abolished subsequent to transfect with the miR-15a-3p and mutant linc00511. The results acquired by qPCR in bladder cancer tissues further demonstrated that the levels of miR-15a-3p and linc00511 were negatively correlated by linear regression analysis. However, whether the interaction of miR-15a-3p and linc00511 could regulate the expression of Bcl-xl downstream and the EMT process and further impact on tumor progression need our future explore.

Wnt/β-catenin signaling pathway regulates various biological events in the cell, including cell growth, apoptosis and metastasis. Previous study indicated that Wnt/β-catenin signaling pathway played crucial role in bladder cancer occurrence and progression [34–37]. Zhijun et al. [35] have proved lncRNA CASC2 could regulate tumor progression through the activation of the Wnt/β-catenin signaling pathway in bladder cancer. To accurately clarify the mechanism of the inhibited cell growth and migration which induced by the knockdown of linc00511 in bladder cancer cells, we explored the effects of linc00511 on Wnt/β-catenin signaling pathway. The results showed that knockdown of linc00511 could inhibit the genes and proteins levels of β-catenin and downstream target genes expression of cyclin D1 and c-myc. However, these effects were reversed by Wnt/β-catenin signal pathway activator SKL2001. According to our results, we considered linc00511 might be associated with the Wnt/β-catenin signaling pathway, which provided a new clue to the pathogenesis of bladder cancer.

In summary, our study illustrated that the linc00511 was highly expressed in tumor tissues and cell lines of bladder cancer. The linc00511 silence could inhibit the bladder cancer cell proliferation, migration and promote cell apoptosis of the bladder cancer cells. In addition, the carcinogenic effects mediated by linc00511 were partially through binding with another oncogenicmiRNA-miR-15a-3p, or via regulating the activation of Wnt/β-catenin signaling pathway. Our results provided insights into the potential mechanism of linc00511 in bladder cancer carcinogenesis and indicated that linc00511 might be a potential diagnosis and a target for bladder cancer treatment. However, more in-depth experiments to explore the underlying mechanism of linc00511 need to be further study.

## Abbreviations

GAS5: growth arrest-specific 5
linc00511: intergenic noncoding RNA 00511
LncRNA: long non-coding RNA
NSCLC: non-small-cell lung cancer
TBST: Tris buffered saline tween
